# Comparative Study of the Influence of Heat Treatment on Fracture Resistance of Different Ceramic Materials Used for CAD/CAM Systems

**DOI:** 10.3390/ma17061246

**Published:** 2024-03-08

**Authors:** Andrea Ordoñez Balladares, Cristian Abad-Coronel, Joao Carlos Ramos, Jorge I. Fajardo, Cesar A. Paltán, Benjamín José Martín Biedma

**Affiliations:** 1 Faculty of Dentistry, Universidad Bolivariana del Ecuador, Durán 092406, Ecuador; adordonezb@ube.edu.ec; 2 Faculty of Dentistry, Universidad de Guayaquil, Guayaquil 090514, Ecuador; 3 Faculty of Dentistry, University of Santiago de Compostela, 15782 Galicia, Spain; benjamin.martin@usc.es; 4 Digital Dentistry and CAD/CAM Materials Research Group, Faculty of Dentistry, Universidad de Cuenca, Cuenca 010107, Ecuador; 5 Faculty of Dentistry, Universidad San Francisco de Quito, Quito 170901, Ecuador; 6 Faculty of Medicine, University of Coimbra, 300-370 Coimbra, Portugal; joao.ramos@ipmd.pt; 7 New Materials and Transformation Process Research Group GiMaT, Universidad Politécnica Salesiana, Cuenca 010102, Ecuador; jfajardo@ups.edu.ec (J.I.F.); cpaltan@ups.edu.ec (C.A.P.)

**Keywords:** ceramics, crystallization, mechanical characterization, CAD/CAM materials, properties, thermal influence

## Abstract

The aim of this study was to compare the influence of heat treatment on fracture resistance (FR) of different ceramic materials used for CAD/CAM systems. Methods: Eighty monolithic restorations were designed using the same parameters and milled with a CAD/CAM system (CEREC SW 5.0, PrimeMill, Dentsply-Sirona™, Bensheim, Germany), forming five study groups: Group 1 (n = 10), CEREC Tessera (Dentsply-Sirona™, Bensheim, Germany) crystallized (CCT), Group 2 (n = 10), CEREC Tessera uncrystallized (UCT), Group 3 (n = 20), Emax-CAD (Ivoclar Vivadent, Schaan, Liechtenstein) (CEC), Group 4 (n = 20), Vita Suprinity (Vita Zahnfabrik, Bad Säckingen, Germany) (CVS), and Group 5 (n = 20) Cameo (Aidite, Qinhuangdao, China) (CC). Results: The average FR was similar for CCT, CC, and CEC at above 400 N, while CVS and UCT had the lowest values at 389,677 N and 343,212 N, respectively. Conclusion: Among the three ceramic materials that exhibited an FR above 400 N, CCT was considered the first recommended choice for CAD/CAM systems. This material not only demonstrated the highest FR but also exhibited outstanding consistency in the related measurements without the presence of outliers. Although the CC material showed high FR, its high dispersion revealed inconsistencies in the repetitions, suggesting caution in its use.

## 1. Introduction

The emphasis on esthetics in dental restorations has concomitantly driven the rapid evolution of metal-free ceramic materials [1,2], used and valued for their outstanding mechanical and optical properties regarding dental restorations [3,4,5,6]. As an example, lithium silicate-based (SL) glass ceramics designed for dental CAD/CAM systems have been introduced in the market [7]. These SL-based ceramics are mainly composed of Li_2_O and SiO_2_. Depending on the predominant phase during their crystallization, they are classified as “lithium disilicate” (Li_2_SiO_25_), “lithium silicate” (Li_2_SiO_3_), or “(di)lithium silicate” (the latter regarding those with significant proportions of both former phases) [8,9,10]. In this study, after an exhaustive review of the ceramic materials for CAD/CAM systems currently available in the dental field, they were classified into four types as detailed in Table 1 and associated into five study groups; see Table 2.

There is an inverse relationship between mechanical and optical properties. Materials with higher crystalline content are characterized by higher mechanical properties, but higher opacity, while higher vitreous content results in higher translucency, but is characterized by lower mechanical performance. It should be considered that translucency is critical in material selection and is of great clinical importance, also it is advantageous for materials to have ideal mechanical performance, so the field is constantly evolving [11,12].

When LDS ceramics are produced for restorative use, additional steps are required for their milling, such as crystallization, because there are small crystals that, when exposed to heat, enlarge and form colonies that intertwine among themselves. This causes material microstructure similar to a mesh, resulting in a dentritic morphology in the form of a tree or sheaf with important ramifications. These acicular structures help to achieve high resistance and tenacity to fracture in glass–ceramic [13,14,15].

One of the most recent iterations of glass matrix ceramics is called CEREC Tessera™ (Dentsply Sirona, Germany). It is characterized as an advanced SL with a glass content of 40–45% and a submicron particle size of ~0.5 μm; it is composed of ~40% lithium disilicate crystals, 5% lithium phosphate and 5% virgilite crystals, which are small (<100 nm) lithium aluminum silicate crystals present in the form of platelets. When using this material, the manufacturer recommends subsequent milling and surface glazing, in addition to subjecting the restoration to heat treatment AHT of 4 min and 30 s to optimize the crystalline structure by forming new virgillite crystals; this nucleation guarantees an increase in its mechanical properties, 700 MPa FR, and better physical properties by maximizing the presence of crystals and the generation of compressive stress around them [16,17,18].

The inclusion of virgillite crystals, proposed to improve translucency [19], suggests the manufacturer to consider a UCT variant with the aim of improving machinability during milling. It is crucial to point out the importance of high precision in this process, as low precision could lead to errors in dental prostheses, generating marginal discrepancies between the crown and the tooth, potentially leading to clinical failure [14,20].

The FR of a metal-free ceramic material is determined by the analysis of its mechanical properties. To elaborate, the masticatory loads in the oral cavity of humans act with various dimensions and directions [21,22,23]. Thus, ceramic materials required to withstand these loads experience forces including compression, tension, and shear. Such varying stresses result in more complex loading patterns and, hence, it is possible for a restorative material to fail even under lower-than-expected loads. Therefore, FR stands out as a factor integral to the determination of the longevity of restorations [24,25,26,27]. Indeed, it is essential to understand that FR is intrinsically linked to the composition and microstructure of a given dental restorative material [28,29].

It is therefore important to examine the abovementioned mechanical properties of a restorative material under thermal influence in a furnace recommended by the manufacturer and under the given AHT program. Notably, incorrect temperature increases or mismanagement of the cooling rate of a material could influence both the mechanical and optical characteristics of an AHT program [30,31]. Moreover, the internal fit and marginal accuracy could be affected by the crystallization of crystalline-reinforced ceramic materials [32,33,34,35].

One of the objectives of the digital flow is to optimize clinical time and maximize the possibility of finishing a restoration in the course of a single appointment; thus, the time spent in the treatment of the restorative material is a variable that should be discussed and considered. Clearly, the less time taken to fabricate CAD/CAM ceramic restorations without affecting the mechanical properties, the more efficient the restorative process [36,37,38,39,40]. In this light, the aim of this study is to compare the influence of heat treatment on the fracture toughness of different ceramic materials used in CAD/CAM systems. The null hypothesis of this research states that there will be no significant differences in the fracture toughness of different ceramic materials used in CAD/CAM systems when subjected to different heat treatments.

## 2. Materials and Methods

### 2.1. Sample Processing

**Figure 1:**
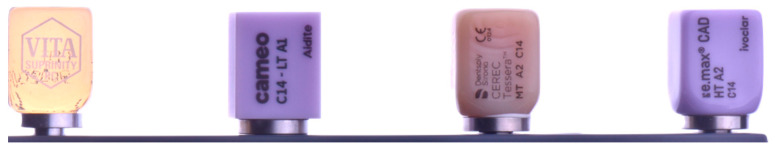
CAD/CAM ceramic blocks.

**Figure 2:**
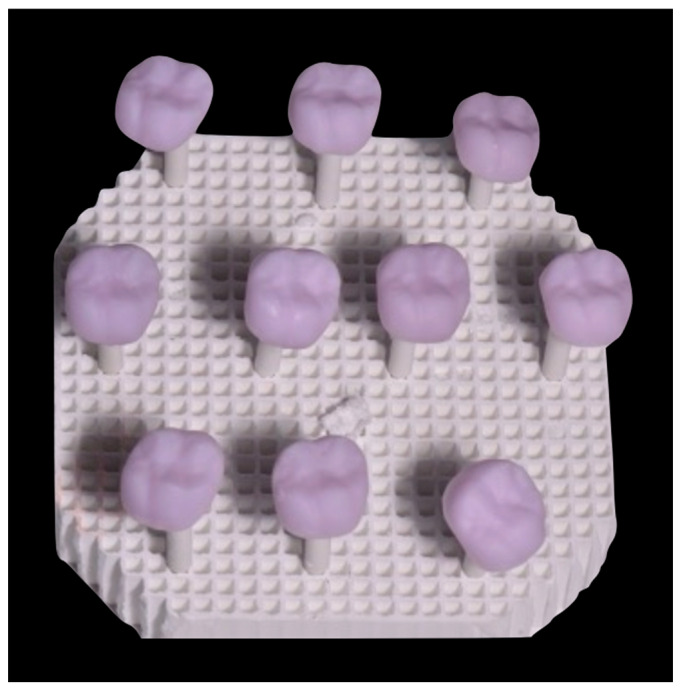
CAD/CAM/SC ceramic blocks.

**Figure 3:**
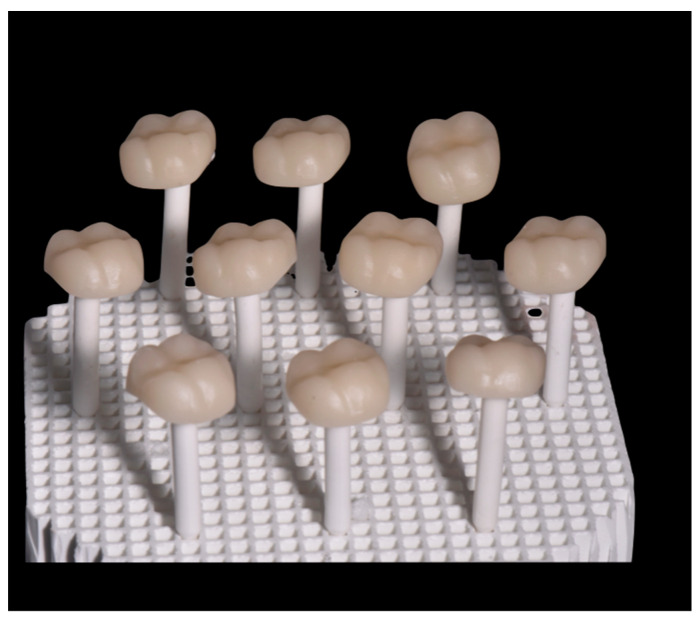
CAD/CAM/C ceramic blocks.

To prepare the samples, the model of an upper first molar designed for a full crown with a chamfer finish line was scanned for this study. The preparation was digitized using an intraoral structured light scanner (Primescan, Dentsply Sirona™, Bensheim, Germany). A full-volume restoration of 1.25 mm thickness was designed using the CAD software (CEREC SW 5.0, CEREC, Dentsply Sirona™, Bensheim, Germany); thereafter, the prepared blocks (Figure 1) were machined using a milling machine (PrimeMill, Dentsply Sirona™, Bensheim, Germany) and subjected to the manufacturer-recommended heat treatment (as detailed in Table 3) and (Figure 2 and Figure 3), eventually forming five study groups (Table 2).

The number of samples was based on various articles and assays used for this purpose [41,42,43,44,45].

### 2.2. Thermocycling

All samples were subjected to a thermocycling process; in total, 5000 [6] cycles were used to estimate five years of oral conditions. Thermocycling was programed with temperature extremes of 5 °C and 55 °C in distilled water (residence time: 25 s; pause time: 10 s) and performed on the computerized thermocycling unit (Thermocycler™, SD Mechatronik, Westerham, Germany).

### 2.3. Fracture Resistance Test

**Figure 4:**
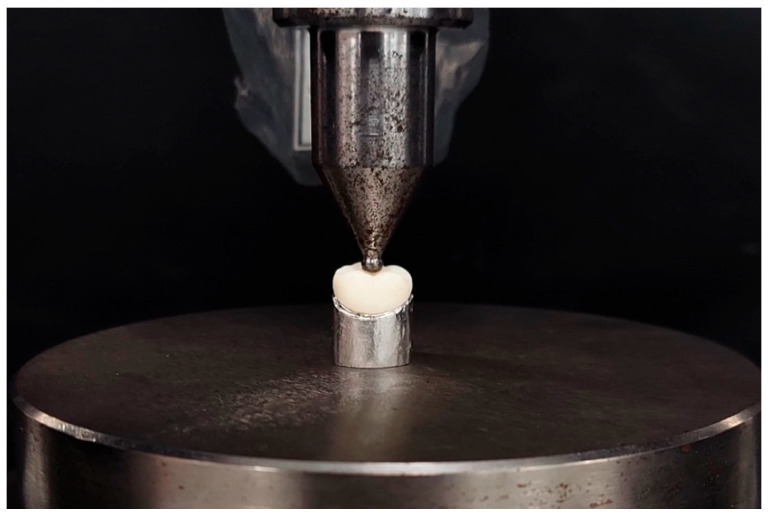
Loading of the specimen seated on the cobalt–chromium alloy abutment using a universal testing machine.

A cast-metal master die obtained from the study’s initial scan of the original typodont was fabricated to support the try-in of each ceramic restoration specimen. Each specimen was subjected to a static load test at a speed of 0.5 mm/min in a direction parallel to the major axis of the tooth and with an initial preload of 10 Newtons (N) using a universal testing machine (Shimadzu, AGS X Series, Tokyo, Japan) equipped with a 20 kN load cell. The load was applied using a hardened-steel pilot punch with a radius of 3 mm in the central pit of the restoration. All specimens were loaded to fracture and recorded in Newtons (N) by a software (Trapezium X Testing Software, Shimadzu, Tokyo, Japan) connected to the testing machine (Figure 4).

### 2.4. Fracture Mode Evaluation

After loading, the fracture surfaces of the specimens were observed and analyzed using a high-resolution stereomicroscope [Olympus; SZX7, New York, NY, USA].

### 2.5. Data Analysis

Data from each of the abovementioned groups were recorded in an Excel™ spreadsheet (Microsoft, Redmond, WA, USA). Subsequently, they were imported into a database in the SPSS 22 software (Statistical Package for Social Science, IBM Corporation, New York, NY, USA) for descriptive and inferential statistical analyses. For these purposes, nonparametric tests, such as the Kruskal–Wallis test, were used. To further explore the differences in data between the groups, multiple comparisons were performed using the Mann–Whitney U statistic.

## 3. Results

**Figure 5:**
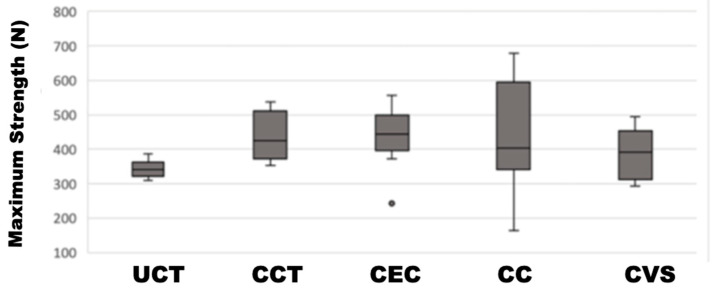
Box-and-whisker plot representing the data of the groups studied.

The descriptive statistics revealed that the maximum strain was reached by material CCT of 437,462 ± 69.17, followed by material CC 436,604 ± 161.403. They were followed by material CEC with a maximum strain of 434,968 ± 88.019 and moderate dispersion. In the fourth position came material CVS, which recorded a maximum strain of 389,677 ± 73.85. Finally, the material with the lowest maximum strain was UCT 343,212 ± 25.143 (Figure 5).

To further explore these differences that were observed, multiple comparisons were carried out using the Mann–Whitney U statistic, as evidenced in Table 4. This study found that there were statistically significant differences in terms of maximum compression between the UCT material and the CC, CCT and CEC materials (*p*-value < 0.05).

**Figure 6:**
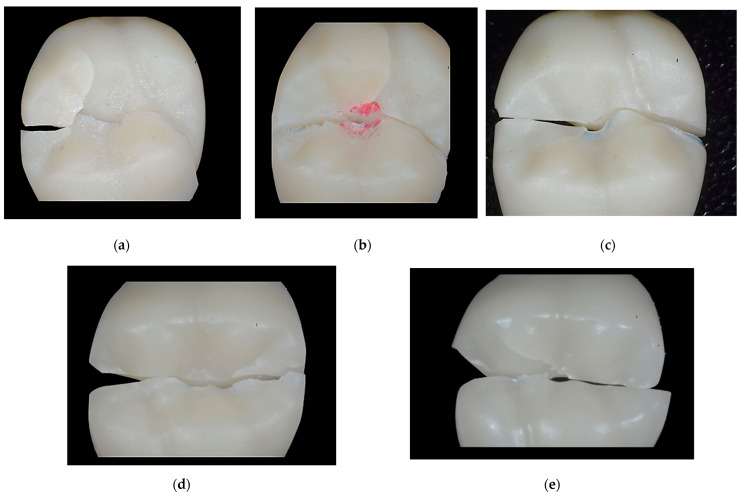
Images of the fracture surfaces concomitant to the five materials studied: (**a**) UCT (**b**) CCT, (**c**) CVS, (**d**) CEC, (**e**) CC.

A fractographic analysis was carried out (Figure 6), where it was visualized that there was a small plastic deformation at the site of the load application, in which there was absorption of the deformation energy; this in Figure 6a,c. However, a zig zag brittle fracture typical of materials with crystalline structures occurred. The cracks followed the grain boundaries with irregular routes as seen in Figure 6b,d,e. These materials require greater strength for fracture due to their crystalline structure.

## 4. Discussion

Glass ceramics (GC) must be properly crystallized according to the manufacturer’s instructions [5,46]. In the case of GC, an inadequate crystallization process may not only negatively influence their mechanical and optical properties [24,47], but also have a negative effect on their mechanical and optical properties [33,48] and increase the marginal gap as well [49]. Considering these aspects, this study aimed to compare the influence of heat treatment on the FR of different ceramic materials used in CAD/CAM systems.

The control group consisted of CCT that, upon receiving AHT following the manufacturer’s indications, optimized its crystalline structure, improving its mechanical properties due to the high content of submicron particles of 0.5 μm, ~40% lithium disilicate crystals, 5% lithium phosphate, and finally 5% of small lithium aluminum silicate crystals interlocked in a glassy matrix enriched with zirconium [16,18,50]. Moreover, among the analyzed properties of the studied materials was the compressive strength (CR), an important parameter involved in determining the mechanical behavior of brittle materials [50]. As mentioned before, based on the findings of this study, the null hypothesis was rejected as significant differences were found between the groups of ceramics studied (*p* < 0.05).

As shown in Table 4, the CCT samples had higher values of force (437.462 N) compared to UCT (343.212 N). These results are in agreement with those of several authors, e.g., Riquieri et al. [51], who characterized the microstructure and evaluated the mechanical properties of two GC before and after AHT; Celtra© Duo (CD) had a higher FR of 251.25 MPa after crystallization, illustrating that AHT develops a fine, dense, homogeneous, and polish-resistant microstructure in the given material. Other studies assure that polishing performed manually generates more compressive stresses, creating more significant microstructural defects in those ceramics that are not subjected to AHT [28,52,53].

On the other hand, CEC obtained a response to FR of 434,968 N, similar to that of CCT. These results simply continue confirming the conclusions of previous studies, demonstrating that GC receiving AHT (following the manufacturer’s indications) show significantly improved FR [54,55]. Importantly, a change in protocol could alter the mechanical behavior of ceramics [56]. For example, the cooling step is a sensitive protocol that must be controlled because lithium disilicate ceramics have a monoclinic, orthorhombic crystalline structure and are therefore anisotropic [57]. The mechanical properties of these brittle materials depend on the residual stresses that can develop due to thermal stresses and phase transformation, which can cause distortion, decreasing the fracture toughness due to the crystallographic orientation of each grain [58]. Prolonged heat treatment (THP) also has a negative influence on this orientation. In fact, an in vitro study performed by Schweitzer [47] evaluated the influence of THP on the FR of two GC with lithium dislylate and lithium silicate (CEC and CD); the FR of CEC increased considerably (to 344.82 MPa), as it received higher temperature during crystallization. Further, AHT is of particular interest with regard to the final composition of GC. Both the crystalline Li_2_Si20_5_ and the residual amorphous phase are responsible for the mechanical properties and optical characteristics of the GC. In the CEREC material, Tessera™, in addition to the elongated configuration of Li_2_Si20_5_, the crystalline phase also contains virgillite crystals LiAlSiO_26_ embedded in a zirconium-enriched glass matrix, conferring to the material a higher density and therefore high FR [59,60]. Material manufacturers report that the addition of zirconia crystals increases the strength of GC [61]. However, a previous study claimed the opposite, stating that there are no clinical advantages for zirconia-reinforced LiO-SiO_22_ [62]. A possible explanation behind this impasse could be the percentage of zirconium: a less than 5% ZrO_2_ content presents a 70% crystalline phase in the amorphous matrix, whereas a more than 10% ZrO_2_ reinforcement reduces the crystalline phase to 40% [63,64,65], significantly affecting the mechanical properties of a given GC. These assertions are supported by this study as herein, CSC displayed the lowest force values of (389.677 N). Although CC yielded “higher” results regarding force (436.604 N), its variability was higher; for this reason, more studies are suggested to support these CC results. In another study that focused on analyzing the influence of AHT on the mechanical behavior of four types of CAD-CAM lithium silicate GC, CEC and Rosetta^®^ SM (RSM) showed higher FR than lithium silicate ceramics with zirconia; in addition, CEC presented a higher Weibull modulus, certifying that this ceramic is more homogeneous and dense [66]. Other authors evaluated and compared the mechanical properties of nine CAD/CAM materials: in their study, after receiving AHT, CEC displayed significantly higher values (*p* < 0.001), with a minimum of 1.69, maximum of 2.46, and a standard deviation of 2.18 [67].

The evaluation and correlation of FS with FR is revealed as a crucial parameter to determine the mechanical strength of brittle materials GC [28], as evidenced in previous studies. The results suggest that materials subjected to AHT might be suitable for PFP as supported by previous research. In contrast, those that have not undergone AHT might be suitable for unitary fixed prostheses (UFP), as supported in a study by Vichi et al. comparing the FS of 4 GC which showed that the highest CEC valued were recorded (350 MPa) after receiving AHT [68]. This trend is confirmed in an analysis by Freitas et al. who evaluated the FS of three ceramic groups, highlighting that UCT exhibited the lowest values (215 MPa) [69]. Furthermore, the results of our research align with the findings of Keshmiri et al. who investigated CVS and obtained FS results (347 MPa) [70] very similar to those obtained in our study. Despite the introduction of virgillite crystals that “improve” translucency [71], a study by Mangla et al. shows the opposite: UCT obtained the lowest values of MT (24.677 ± 0.187) and HT (27.447 ± 0.820) when compared to CEC MT (29.366 ± 1.243) and HT (30.771 ± 0.912) [19].

Undoubtedly, one of the benefits of CAD/CAM technology is that it provides an avenue for the rapid production of dental restorations [38,72,73]. However, it also presents challenges inherent in the subtraction process, as milling the ceramic creates microstructural defects in the restorations [74]. In addition, preparation design, crown design, die type, cement space, type of cementing agent, and thickness of the restorations together influence the strength of a ceramic material [75], so they should be evaluated independently. Consequently, 1 mm thick crowns using CCT ceramics are lower, 1911.4 ± (468.4 N), in contrast to those found in CEC (2995.3 ± 880.6 N), regardless of the type of cementation [60]. Hamza et al. examined the FR of different CAD/CAM ceramic materials after applying a self-adhesive cementation protocol. The results obtained were much higher for CVS (1742.9 ± 102.7 N) compared to CEC (1565.2 ± 89.7 N) [76]. For this reason, in this study, we chose not to perform cementation in order to obtain precise and specific information exclusively on the mechanical behavior of CAD/CAM ceramics placed under thermal influence.

The modulus of elasticity provided by the die material is directly related to the mechanical strength of the material. In this regard, one study [76] evaluated the FR of ceramic crowns with respect to the modulus of elasticity of the die; it found that the fracture load increased significantly with increasing modulus of elasticity. Another study applied a compression test to 60 molar crowns composed of various types of CAD/CAM ceramics, cemented on epoxy resin dies, again showing a FR of 3100 N [77]. Several researchers agree with the argument that increasing the modulus of elasticity of the support material can increase the FR of a GC [78,79]. Although Yucel and Yondem [80] stated that ceramic crowns supported on stainless steel dies have higher FR values compared to ceramic crowns supported on dentin dies; they recommended the use of dies composed of materials with low elastic modulus for mechanical testing, simulating the clinical environment more accurately. Although a die material with these characteristics could have been chosen in this study, it is possible that breakage was anticipated earlier than that of the ceramic under study. Therefore, a cobalt–chromium metal support structure was selected in this study.

In the fractographic analysis, it was observed that once the critical stress value was reached, unstable cracks were generated, which facilitated crack propagation until failure occurred without the occurrence of plastic deformation, as expected with respect to ceramic materials. This finding is supported by previous studies [80,81]. In addition, Abad-Coronel et al. conducted a study that analyzed the fracture toughness of materials used in temporary fixed prosthodontics. In their fractographic analysis, they found that acetal resin (AR) was the material that showed the highest percentage of deformation [82].

Considering the limitations of the methodology used in the present investigation and with the objective of obtaining the results of a complete biomechanical behavior of the material used for dental restoration, it is important to carry out future studies replicating the clinical conditions under which this study was performed. This is because thermal and chemical changes and humidity lead to the aging of the ceramic restoration outlined by CAD/CAM systems.

In the future, randomized controlled clinical studies are also needed to validate the biomechanical behaviors of CAD/CAM ceramic restorations, which can replicate various other clinical conditions.

Within the limitations of this study, the exclusive focus on fracture toughness through a compressive test, without addressing other properties such as flexural strength, hardness, and fracture toughness, stands out. In addition, the microstructure of the material was not examined since it was not the main objective of the research. Future studies are urged to incorporate microstructural analyses of ceramics, providing a more complete understanding of changes in the microstructure of ceramic materials after undergoing additional heat treatments.

## 5. Conclusions

In summary, AHT proves to be crucial for improving the mechanical properties of the studied GC with respect to dental restoration:

- In CCT, AHT optimizes the microstructure thanks to its advanced composition and the formation of new virgillite crystals, consequently increasing its FR, thus allowing for it to be resistant to possible fissures and cracks created by subtractive processes during the fabrication of restorations.
- The results given by CE allow for us to conclude that these ceramics have a high capacity of absorbing and distributing stresses without suffering fractures.
- Although CC displays the highest FR values, its high dispersion indicates inconsistencies; one must exercise caution while using it.

Importantly, it is essential to use a crystallization temperature that is suitable with regard to the size of the prosthesis, following the manufacturer’s indications, in order to prevent the incidence of alterations in its mechanical properties.

## Figures and Tables

**Table 1:** Sample and manufacturer details of the four types of CAD/CAM ceramics.

Name of Material	Manufacturer	Lot Number	Composition
Cerec Tessera	Dentsply Sirona™/Germany	16015140	Li_2_Si_2_O_5_: 90% Li_3_PO_4_: 5% Li_0.5_A_l0.5_Si_2.5_O_6_ (virgilite): 5%
Emax CAD	Ivoclar Vivadent™/Liechtenstein	6788824	SiO_2_: 57–80% Li_2_O: 11–19% K_2_O: 0–13% P_2_O_5_: 0–11% ZrO_2_: 0–8% ZnO: 0–8% Coloring oxides: 0–8%
Vita Suprinity	Vita Zahnfabrik/Germany	7971329	SiO_2_: 56–64% Li_2_O: 15–21% ZrO_2_: 8–12% P_2_O_5_: 3–8% K_2_O: 1–4% Al_2_O_3_: 1–4% CeO_2_: 0–4% Pigments: 0–4%
Cameo	Aidite/Singapore	9180451	50–70% SiO_2_; 19–20% Li_2_O; 0–13% K_2_O; 0–11% P_2_O_5_; 0–5% ZrO_2_, 0–8% ZnO; 0–11% others + coloring

**Table 2:** Study groups, ceramics and samples.

Groups	Ceramics	Samples
Group 1	Cerec Tessera (CCT)	10
Group 2	Cerec Tessera (UCT)	10
Group 3	Emax Cad (CEC)	20
Group 4	Suprinity (CVS)	20
Group 5	Cameo (CC)	20

UC: Uncrystallized; C: Crystallized.

**Table 3:** CAD/CAM ceramics and manufacturer-recommended AHT.

Name of Material	Oven	Manufacturer	Heating Units	Time
Cerec Tessera	Programat P310	Ivoclar Vivadent™/ Liechtenstein	760 °C	2 min
Emax CAD			850 °C	7 min
Vita Suprinity			840 °C	8 min
Cameo			840 °C	6 min

**Table 4:** Multiple comparisons: Mann–Whitney U test.

Sample 1–Sample 2	Test Statistic	Median	Sig.
Tessera uncrystallized–Cameo	−14,900	62.677	0.017 *
Tessera uncrystallized–Tessera crystallized	−17,189	83.924	0.008 *
Tessera uncrystallized–E-Max	−17,500	103.863	0.005 *

Note: Significance level of 5%. Shapiro–Wilk: *p*-value ≥ 0.05; Levene’s test: *p*-value < 0.05; Kruskal–Wallis H: *p*-value < 0.05. * *p*-value < 0.05.

## Data Availability

Data are contained within the article.

## References

[1] Choi J.-W., Kim S.-Y., Bae J.-H., Bae E.-B., Huh J.-B. (2017). In Vitro Study of the Fracture Resistance of Monolithic Lithium Disilicate, Monolithic Zirconia, and Lithium Disilicate Pressed on Zirconia for Three-Unit Fixed Dental Prostheses. J. Adv. Prosthodont..

[2] Rada S., Zhang J., Rada R., Culea E. (2022). Advanced Zirconia Ceramics Stabilized with Yttria and Magnesia: Structure and Vickers Microhardness. J. Mech. Behav. Biomed. Mater..

[3] Motro P.F.K., Kursoglu P., Kazazoglu E. (2012). Effects of Different Surface Treatments on Stainability of Ceramics. J. Prosthet. Dent..

[4] May M.M., Fraga S., May L.G. (2022). Effect of Milling, Fitting Adjustments, and Hydrofluoric Acid Etching on the Strength and Roughness of CAD-CAM Glass-Ceramics: A Systematic Review and Meta-Analysis. J. Prosthet. Dent..

[5] Gautam C., Joyner J., Gautam A., Rao J., Vajtai R. (2016). Zirconia based dental ceramics: Structure, mechanical properties, biocompatibility and applications. Dalton Trans..

[6] Ordoñez Balladares A., Abad-Coronel C., Ramos J., Martín Biedma B. (2022). Fracture Resistance of Sintered Monolithic Zirconia Dioxide in Different Thermal Units. Materials.

[7] Rinke S., Zuck T., Hausdörfer T., Leha A., Wassmann T., Ziebolz D. (2022). Prospective Clinical Evaluation of Chairside-Fabricated Zirconia-Reinforced Lithium Silicate Ceramic Partial Crowns—5-Year Results. Clin. Oral Investig..

[8] Abdulrahman S., Von See Mahm C., Talabani R., Abdulateef D. (2021). Evaluation of the clinical success of four different types of lithium disilicate ceramic restorations: A retrospective study. BMC Oral Health.

[9] Munoz A., Zhao Z., Paolone G., Louca C., Vichi A. (2023). Flexural Strength of CAD/CAM Lithium-Based Silicate Glass–Ceramics: A Narrative Review. Materials.

[10] Elsaka S.E., Elnaghy A.M. (2016). Mechanical Properties of Zirconia Reinforced Lithium Silicate Glass-Ceramic. Dent. Mater..

[11] Cokic S.M., Vleugels J., Van Meerbeek B., Camargo B., Willems E., Li M., Zhang F. (2020). Mechanical Properties, Aging Stability and Translucency of Speed-Sintered Zirconia for Chairside Restorations. Dent. Mater..

[12] Kontonasaki E., Giasimakopoulos P., Rigos A.E. (2020). Strength and Aging Resistance of Monolithic Zirconia: An Update to Current Knowledge. Jpn. Dent. Sci. Rev..

[13] Beuer F., Schweiger J., Edelhoff D. (2008). Digital Dentistry: An Overview of Recent Developments for CAD/CAM Generated Restorations. Br. Dent. J..

[14] Kirsch C., Ender A., Attin T., Mehl A. (2017). Trueness of Four Different Milling Procedures Used in Dental CAD/CAM Systems. Clin. Oral Investig..

[15] Yin R., Jang Y.-S., Lee M.-H., Bae T.-S. (2019). Comparative Evaluation of Mechanical Properties and Wear Ability of Five CAD/CAM Dental Blocks. Materials.

[16] Reid D., Matis J., Lien W., Raimondi C., Arnason S., DuVall N., Vandewalle K. (2023). Optical and Mechanical Properties of New Ceramic CAD/CAM Materials. Oper. Dent..

[17] Phark J., Duarte S. (2022). Microstructural Considerations for Novel Lithium Disilicate Glass Ceramics: A Review. J. Esthet. Restor. Dent..

[18] Hurle K., Lubauer J., Belli R., Lohbauer U. (2022). On the Assignment of Quartz-like LiAlSi_2_O_6_-SiO_2_ Solid Solutions in Dental Lithium Silicate Glass-Ceramics: Virgilite, High Quartz, Low Quartz or Stuffed Quartz Derivatives?. Dent. Mater..

[19] Mangla P. (2022). Measurement of Translucency, Biaxial Flexural Strength, and Radiopacity of Different Lithium Disilicate Materials. Master’s Thesis.

[20] Bosch G., Ender A., Mehl A. (2014). A 3-Dimensional Accuracy Analysis of Chairside CAD/CAM Milling Processes. J. Prosthet. Dent..

[21] Badawy R., El-Mowafy O., Tam L.E. (2016). Fracture Toughness of Chairside CAD/CAM Materials–Alternative Loading Approach for Compact Tension Test. Dent. Mater..

[22] Amesti-Garaizabal A., Agustín-Panadero R., Verdejo-Solá B., Fons-Font A., Fernández-Estevan L., Montiel-Company J., Solá-Ruíz M.F. (2019). Fracture Resistance of Partial Indirect Restorations Made with CAD/CAM Technology. A Systematic Review and Meta-Analysis. J. Clin. Med..

[23] Guarda G., Correr A., Gonçalves L., Costa A., Borges G., Sinhoreti M., Correr-Sobrinho L. (2013). Effects of Surface Treatments, Thermocycling, and Cyclic Loading on the Bond Strength of a Resin Cement Bonded to a Lithium Disilicate Glass Ceramic. Oper. Dent..

[24] Seghi R., Denry I., Rosenstiel S. (1995). Relative Fracture Toughness and Hardness of New Dental Ceramics. J. Prosthet. Dent..

[25] Sonmez N., Gultekin P., Turp V., Akgungor G., Sen D., Mijiritsky E. (2018). Evaluation of Five CAD/CAM Materials by Microstructural Characterization and Mechanical Tests: A Comparative in Vitro Study. BMC Oral Health.

[26] Walker P.D., Ruse N.D. (2019). “CAD-on” Interfaces–Fracture Mechanics Characterization. J. Prosthodont..

[27] Elraggal A., Afifi R., Abdelraheem I. (2022). Effect of Erosive Media on Microhardness and Fracture Toughness of CAD-CAM Dental Materials. BMC Oral Health.

[28] Wendler M., Belli R., Petschelt A., Mevec D., Harrer W., Lube T., Danzer R., Lohbauer U. (2017). Chairside CAD/CAM Materials. Part 2: Flexural Strength Testing. Dent. Mater..

[29] Hong M.-S., Choi Y.-S., Lee H.-H., Lee J.-H., Ahn J. (2021). Comparison of Mechanical Properties of Chairside CAD/CAM Restorations Fabricated Using a Standardization Method. Materials.

[30] Lohbauer U., Belli R., Alonso A.A., Goetz-Neunhoeffer F., Hurle K. (2019). Effect of Sintering Parameters on Phase Evolution and Strength of Dental Lithium Silicate Glass-Ceramics. Dent. Mater..

[31] Nejatidanesh F., Azadbakht K., Savabi O., Sharifi M., Shirani M. (2020). Effect of Repeated Firing on the Translucency of CAD-CAM Monolithic Glass-Ceramics. J. Prosthet. Dent..

[32] Vasiliu R.-D., Porojan S.D., Porojan L. (2020). In Vitro Study of Comparative Evaluation of Marginal and Internal Fit between Heat-Pressed and CAD-CAM Monolithic Glass-Ceramic Restorations after Thermal Aging. Materials.

[33] Azarbal A., Azarbal M., Engelmeier R.L., Kunkel T.C. (2018). Marginal Fit Comparison of CAD/CAM Crowns Milled from Two Different Materials. J. Prosthodont..

[34] Kim J.-H., Oh S., Uhm S.-H. (2016). Effect of the Crystallization Process on the Marginal and Internal Gaps of Lithium Disilicate CAD/CAM Crowns. BioMed Res. Int..

[35] Falahchai M., Ghavami-Lahiji M., Rasaie V., Amin M., Neshandar Asli H. (2023). Comparison of Mechanical Properties, Surface Roughness, and Color Stability of 3D-Printed and Conventional Heat-Polymerizing Denture Base Materials. J. Prosthet. Dent..

[36] Stanley M., Paz A.G., Miguel I., Coachman C. (2018). Fully Digital Workflow, Integrating Dental Scan, Smile Design and CAD-CAM: Case Report. BMC Oral Health.

[37] Sailer I., Benic G.I., Fehmer V., Hämmerle C.H., Mühlemann S. (2017). Randomized Controlled Within-Subject Evaluation of Digital and Conventional Workflows for the Fabrication of Lithium Disilicate Single Crowns. Part II: CAD-CAM versus Conventional Laboratory Procedures. J. Prosthet. Dent..

[38] Joda T., Zarone F., Ferrari M. (2017). The Complete Digital Workflow in Fixed Prosthodontics: A Systematic Review. BMC Oral Health.

[39] Blatz M.B., Conejo J. (2019). The Current State of Chairside Digital Dentistry and Materials. Dent. Clin..

[40] Da Silva L.H., de Lima E., Miranda R.B.d.P., Favero S.S., Lohbauer U., Cesar P.F. (2017). Dental Ceramics: A Review of New Materials and Processing Methods. Braz. Oral Res..

[41] Saleh A.R.M., Al-Ani M., ALRawi T., Al-Edressi G. (2021). An In-Vitro Comparison of Fracture Resistance of Three CAD/CAM Ceramic Materials for Fabricating Veneer. Saudi Dent. J..

[42] Blunck U., Fischer S., Hajtó J., Frei S., Frankenberger R. (2020). Ceramic Laminate Veneers: Effect of Preparation Design and Ceramic Thickness on Fracture Resistance and Marginal Quality in Vitro. Clin. Oral Investig..

[43] Heidari N., Amawi R., Seweryniak P., Bakitian F., Vult von Steyern P. (2022). Fracture Resistance and Fracture Behaviour of Monolithic Multi-Layered Translucent Zirconia Fixed Dental Prostheses with Different Placing Strategies of Connector: An in Vitro Study. Clin. Cosmet. Investig. Dent..

[44] Zacher J., Bauer R., Hanie Krifka S., Rosentritt M. (2021). In Vitro Performance and Fracture Resistance of Pressed or CAD/CAM Milled Ceramic Implant-Supported Screw-Retained or Cemented Anterior FDPs. J. Prosthodont. Res..

[45] Abad-Coronel C., Ordoñez Balladares A., Fajardo J.I., Martín Biedma B.J. (2021). Resistance to Fracture of Lithium Disilicate Feldspathic Restorations Manufactured Using a CAD/CAM System and Crystallized with Different Thermal Units and Programs. Materials.

[46] Denry I., Goudouri O., Harless J.D., Hubbard E., Holloway J.A. (2018). Strontium-releasing Fluorapatite Glass-ceramics: Crystallization Behavior, Microstructure, and Solubility. J. Biomed. Mater. Res. Part B Appl. Biomater..

[47] Cattell M.J., Patzig C., Bissasu S., Tsoutsos A., Karpukhina N. (2020). Nucleation Efficacy and Flexural Strength of Novel Leucite Glass-Ceramics. Dent. Mater..

[48] Gold S.A., Ferracane J.L., da Costa J. (2018). Effect of Crystallization Firing on Marginal Gap of CAD/CAM Fabricated Lithium Disilicate Crowns. J. Prosthodont..

[49] Lubauer J., Belli R., Peterlik H., Hurle K., Lohbauer U. (2022). Grasping the Lithium Hype: Insights into Modern Dental Lithium Silicate Glass-Ceramics. Dent. Mater..

[50] Rosentritt M., Hahnel S., Engelhardt F., Behr M., Preis V. (2017). In Vitro Performance and Fracture Resistance of CAD/CAM-Fabricated Implant Supported Molar Crowns. Clin. Oral Investig..

[51] Riquieri H., Monteiro J.B., Viegas D.C., Campos T.M.B., De Melo R.M., De Siqueira Ferreira Anzaloni Saavedra G. (2018). Impact of Crystallization Firing Process on the Microstructure and Flexural Strength of Zirconia-Reinforced Lithium Silicate Glass-Ceramics. Dent. Mater..

[52] Romanyk D.L., Guo Y., Rae N., Veldhuis S., Sirovica S., Fleming G.J., Addison O. (2020). Strength-Limiting Damage and Its Mitigation in CAD-CAM Zirconia-Reinforced Lithium-Silicate Ceramics Machined in a Fully Crystallized State. Dent. Mater..

[53] Kim S.-H., Choi Y.-S., Kang K.-H., Att W. (2022). Effects of Thermal and Mechanical Cycling on the Mechanical Strength and Surface Properties of Dental CAD-CAM Restorative Materials. J. Prosthet. Dent..

[54] Alakkad L., Kostagianni A., Finkelman M., Maawadh A., Ali A., Papathanasiou A. (2021). Biaxial Flexural Strength of Various CAD-CAM Glass-Ceramic Materials. Am. J. Dent..

[55] Simba B.G., Ribeiro M.V., Suzuki P.A., Alves M.F.R.P., Strecker K., Santos C.D. (2019). Mechanical Properties of Lithium Metasilicate after Short-Term Thermal Treatments. J. Mech. Behav. Biomed. Mater..

[56] Serbena F.C., Zanotto E.D. (2012). Internal Residual Stresses in Glass-Ceramics: A Review. J. Non-Cryst. Solids.

[57] Belli R., Lohbauer U., Goetz-Neunhoeffer F., Hurle K. (2019). Crack-Healing during Two-Stage Crystallization of Biomedical Lithium (Di) Silicate Glass-Ceramics. Dent. Mater..

[58] Schweitzer F., Spintzyk S., Geis-Gerstorfer J., Huettig F. (2020). Influence of Minimal Extended Firing on Dimensional, Optical, and Mechanical Properties of Crystalized Zirconia-Reinforced Lithium Silicate Glass Ceramic. J. Mech. Behav. Biomed. Mater..

[59] Lu Y., Dal Piva A.M.O., Nedeljkovic I., Tribst J.P.M., Feilzer A.J., Kleverlaan C.J. (2023). Effect of Glazing Technique and Firing on Surface Roughness and Flexural Strength of an Advanced Lithium Disilicate. Clin. Oral Investig..

[60] Rosentritt M., Schmid A., Huber C., Strasser T. (2022). In Vitro Mastication Simulation and Wear Test of Virgilite and Advanced Lithium Disilicate Ceramics. Int. J. Prosthodont..

[61] Zarone F., Ruggiero G., Leone R., Breschi L., Leuci S., Sorrentino R. (2021). Zirconia-Reinforced Lithium Silicate (ZLS) Mechanical and Biological Properties: A Literature Review. J. Dent..

[62] Pitiaumnuaysap L., Phokhinchatchanan P., Suputtamongkol K., Kanchanavasita W. (2017). Fracture Resistance of Four Dental Computer-Aided Design and Computer-Aided Manufacturing Glass-Ceramics. Mahidol Dent. J..

[63] Hallmann L., Ulmer P., Kern M. (2018). Effect of Microstructure on the Mechanical Properties of Lithium Disilicate Glass-Ceramics. J. Mech. Behav. Biomed. Mater..

[64] Schwindling F.S., Rues S., Schmitter M. (2017). Fracture Resistance of Glazed, Full-Contour ZLS Incisor Crowns. J. Prosthodont. Res..

[65] Monteiro J.B., Riquieri H., Prochnow C., Guilardi L.F., Pereira G.K.R., Borges A.L.S., de Melo R.M., Valandro L.F. (2018). Fatigue Failure Load of Two Resin-Bonded Zirconia-Reinforced Lithium Silicate Glass-Ceramics: Effect of Ceramic Thickness. Dent. Mater..

[66] Corado H.P., da Silveira P.H., Ortega V.L., Ramos G.G., Elias C.N. (2022). Flexural Strength of Vitreous Ceramics Based on Lithium Disilicate and Lithium Silicate Reinforced with Zirconia for CAD/CAM. Int. J. Biomater..

[67] Hampe R., Theelke B., Lümkemann N., Eichberger M., Stawarczyk B. (2019). Fracture Toughness Analysis of Ceramic and Resin Composite CAD/CAM Material. Oper. Dent..

[68] Vichi A., Zhao Z., Paolone G., Scotti N., Mutahar M., Goracci C., Louca C. (2022). Factory Crystallized Silicates for Monolithic Metal-Free Restorations: A Flexural Strength and Translucency Comparison Test. Materials.

[69] Freitas J.S., Souza L.F.B., Pereira G.K.R., May L.G. (2023). Surface Properties and Flexural Fatigue Strength of an Advanced Lithium Disilicate. J. Mech. Behav. Biomed. Mater..

[70] Keshmiri N., Alaghehmand H., Mokhtarpour F. (2020). Effect of Hydrofluoric Acid Surface Treatments on Surface Roughness and Three-Point Flexural Strength of Suprinity Ceramic. Front. Dent..

[71] Matis J.I. (2022). Optical Properties of Novel Ceramic CAD/CAM Materials. Ph.D. Thesis.

[72] Ahlholm P., Sipilä K., Vallittu P., Jakonen M., Kotiranta U. (2018). Digital versus Conventional Impressions in Fixed Prosthodontics: A Review. J. Prosthodont..

[73] Fung L., Brisebois P. (2020). Implementing Digital Dentistry into Your Esthetic Dental Practice. Dent. Clin..

[74] Mota E.G., Smidt L.N., Fracasso L.M., Burnett Jr L.H., Spohr A.M. (2017). The Effect of Milling and Postmilling Procedures on the Surface Roughness of CAD/CAM Materials. J. Esthet. Restor. Dent..

[75] Albelasy E., Hamama H.H., Tsoi J.K., Mahmoud S.H. (2021). Influence of Material Type, Thickness and Storage on Fracture Resistance of CAD/CAM Occlusal Veneers. J. Mech. Behav. Biomed. Mater..

[76] Hamza T.A., Sherif R.M. (2019). Fracture Resistance of Monolithic Glass-ceramics versus Bilayered Zirconia-based Restorations. J. Prosthodont..

[77] Jurado C.A., Lee D., Cortes D., Kaleinikova Z., Hernandez A.I., Donato M.V., Tsujimoto A. (2023). Fracture Resistance of Chairside CAD/CAM Molar Crowns Fabricated with Different Lithium Disilicate Ceramic Materials. Int. J. Prosthodont..

[78] Chen S.E., Park A.C., Wang J., Knoernschild K.L., Campbell S., Yang B. (2019). Fracture Resistance of Various Thickness e. Max CAD Lithium Disilicate Crowns Cemented on Different Supporting Substrates: An in Vitro Study. J. Prosthodont..

[79] Bencun M., Ender A., Wiedemeier D.B., Mehl A. (2020). Fracture Load of CAD/CAM Feldspathic Crowns Influenced by Abutment Material. Materials.

[80] García-Engra G., Fernandez-Estevan L., Casas-Terrón J., Fons-Font A., Castelo-Baz P., Agustín-Panadero R., Román-Rodriguez J.L. (2020). Fracture Resistance of New Metal-Free Materials Used for CAD-CAM Fabrication of Partial Posterior Restorations. Medicina.

[81] Fouda A.M., Atta O., Özcan M., Stawarczyk B., Glaum R., Bourauel C. (2023). An Investigation on Fatigue, Fracture Resistance, and Color Properties of Aesthetic CAD/CAM Monolithic Ceramics. Clin. Oral Investig..

[82] Abad-Coronel C., Calle C., Abril G., Paltán C.A., Fajardo J.I. (2023). Fracture Resistance Analysis of CAD/CAM Interim Fixed Prosthodontic Materials: PMMA, Graphene, Acetal Resin and Polysulfone. Polymers.

